# Long-term effects of a combination of D-penicillamine and zinc salts in the treatment of Wilson’s disease in children

**DOI:** 10.3892/etm.2013.971

**Published:** 2013-02-22

**Authors:** HONG CHANG, AIJING XU, ZHIHONG CHEN, YING ZHANG, FEI TIAN, TANG LI

**Affiliations:** 1 Department of Pediatrics, Affiliated Hospital of Medical College, Qingdao University, Qingdao 266003;; 2 Children’s Hospital, Guangzhou Women and Children’s Medical Center, Guangzhou Medical College, Guangzhou 510520, P.R. China

**Keywords:** Wilson’s disease, D-penicillamine, zinc sulfate, child

## Abstract

The aim of this study was to investigate the effectiveness of a high-dose zinc sulfate and low-dose D-penicillamine combination in the treatment of pediatric Wilson’s disease (WD). A retropective chart review of 65 patients with WD was conducted. These patients received D-penicillamine (8–10 mg/kg/day) and zinc sulfate as the primary treatment. The pediatric dose of elemental zinc is 68–85 mg/day until 6 years of age, 85–136 mg/day until 8 years of age, 136–170 mg/day until 10 years of age and then 170 mg/day, in 3 divided doses 1 h before meals. After clinical and biochemical improvement or stabilization, zinc sulfate alone was administered as the maintenance therapy. Under treatment, the majority of patients (89.2%) had a favourable outcome and 3 patients succumbed due to poor therapy compliance. No penicillamine-induced neurological deterioration was noted and side-effects were observed in <11% of patients over the entire follow-up period. Benefical results on the liver and neurological symptoms were reported following extremely long-term treatment with a combination of low-dose D-penicillamine and high-dose zinc sulfate. Therefore, this regimen is an effective and safe treatment for children with WD.

## Introduction

Wilson’s disease (WD) is an inborn error of copper metabolism caused by autosomal recessive inheritance. This rare disease is caused by mutations in the gene encoding a copper-transporting P-type ATPase (ATP7B), which is important for copper excretion into bile, leading to copper accumulation, mainly in the liver, as well as in the brain, cornea and kidney (1). The estimated prevalence of WD is between 1/30,000 and 1/100,000 individuals; however, its exact incidence has not yet been determined in China. Clinical presentation demonstrates remarkable variability; patients present with progressive liver degeneration, neuropsychiatric symptoms or both. The manifestations are more likely to be hepatic in young children and neurological in adolescents, although other forms of clinical presentation have also been documented (2). In children, the disease usually presents after 3 years of age with either incidental discovery of abnormal liver function tests or as chronic liver disease (3).

Treatment of WD is based on several chelating agents (D-penicillamine and trientine) and zinc salts for medical therapy (4). In general, the approach for treatment is dependent on whether the patient is symptomatic or asymptomatic and on the predominant manifestation of the symptoms (neurological or hepatic). Although it is clearly effective, D-penicillamine has serious toxicity. The side-effects include immunological conditions (lupus-like reactions, nephrotic syndrome, myasthenia gravis and Goodpasture syndrome), skin defects (degenerative changes and elastosis perforans serpiginosa) and joint disorders (arthropathy). It has been reported that 10–50% of D-penicillamine-treated WD patients develop worsening of the neurological manifestations during the initial phase of treatment (5–7). Given these side-effects, trientine and zinc are being increasingly used as preferred or second-line treatment.

Trientine shares a number of the side-effects of penicillamine; however, it appears to be significantly less toxic and as efficacious as penicillamine (8). Tetrathiomolybdate is possibly a better choice than trientine in patients with neurological WD; however, further studies are required (9). Zinc is often used as the initial, first-line monotherapy in asymptomatic or presymptomatic patients and the use of zinc is associated with few side-effects; however, there is still controversy with regard to neurological diseases. It is difficult to confirm which therapy is more effective.

To reduce the side-effects of D-penicillamine and improve the efficacy in the treatment of WD, in 1970 we tested the combination of low-dose D-penicillamine and high-dose zinc sulfate. The patients who had symptoms were treated with high-dose zinc sulfate (100–150 mg, aged <6 years; 150–200 mg, 6–8 years; 200–300 mg, 9–10 years; 300 mg, >10 years; 3 times a day) in addition to low-dose penicillamine (8–10 mg/kg/day) at the beginning of treatment. Zinc sulfate alone was administered to the presymptomatic patients and was used as maintenance therapy when clinical improvement was obtained. We identified that the combined therapy is an effective, safe and cheap treatment for children with WD. We first reported on the combination of low-dose D-penicillamine and high-dose zinc sulfate in children in 1985 (10,11). In the present study, we evaluated the clinical and laboratory findings, treatment and long-term follow-up of WD patients. The objective of our retrospective study was to identify the effectiveness and safety of the combined therapy.

## Patients and methods

### Patients

Clinical and laboratory data were obtained from a review of the files of patients admitted between 1990 and 2008 at the Affiliated Hospital of Qingdao Medical College, China. A total of 89 patients with WD were diagnosed, and 65 of these patients were followed and treated with D-penicillamine combined with zinc sulfate or with zinc sulfate alone. The diagnosis was established on the basis of characteristic clinical manifestations, positive family history and the presence of Kayser-Fleischer (K-F) rings, low ceruloplasmin levels (<200 mg/l), increased 24-h urinary copper excretion (>100 *μ*g/24 h) and urinary excretion in the penicillamine challenge >1600 *μ*g copper/24 h (>25 *μ*moles/24 h). This study was conducted in accordance with the Declaration of Helsinki and with approval from the Ethics Committee of the Affiliated Hospital of Medical College, Qingdao University. Written informed consent was obtained from all participants.

Examination for K-F rings was performed by slit-lamp examination by ophthalmologists. Standard methods were used for liver function tests and other routine laboratory investigations. Eight patients underwent ultrasound-guided liver biopsy. Brain magnetic resonance imaging (MRI) or computed tomography (CT) was performed in 26 patients at the time of diagnosis.

Genetic analysis, available in our center after 1999, was performed on patients and their families. Screening for the Arg778leu mutations in exon 8 of ATP7B was performed in 15 patients.

### Treatment

Symptomatic patients were initially treated with a combination of D-penicillamine and zinc sulfate. The daily dose of D-penicillamine (8–10 mg/kg) was administered before food in two divided doses per day. The pediatric dose of elemental zinc is 68–85 mg/day until 6 years of age, 85–136 mg/day until 8 years of age, 136–170 mg/day until 10 years of age and then 170 mg/day, in three divided doses, 1 h before meals. After clinical and biochemical improvement or stabilization, zinc sulfate only was administered as the maintenance therapy. The drug was adjusted individually according to urinary copper excretion. All patients were placed on a copper-reduced diet.

### Follow-up

Patients were followed up at 1, 3, 6 and 12 months after the procedure and annually afterwards. Laboratory tests, including blood counts, urinalysis, liver biochemistries and copper and zinc metabolism were checked regularly.

### Statistical analysis

Statistical software SPSS version 17.0 (SPSS, Chicago, IL, USA) was used to evaluate data. Differences among groups were compared using one-factor ANOVA. For comparison of the two groups the LSD test was used. P<0.05 was considered to indicate a statistically significant result.

## Results

### Baseline patient characteristics

Sixty-five children (32 males and 33 females) were included in the study group. Clinical presentation was hepatic in 44 patients, purely neurologic in 6 patients and mixed hepatic and neurologic in 15 patients. The modes of presentation are listed in Table I. The mean age of the patients was 9.0 years (range, 5–13 years). Onset with hepatic signs (7.3 years) was earlier than onset with neurological signs (9.8 years). Abdominal symptoms and signs were detected in 59 out of the 65 patients. Among the symptoms, hepatomegaly (76.9%), abnormal liver enzymes (84.6%) and jaundice (18.5%) were the most common findings. Other symptoms included fatigue, edema, ascites and abdominal pain. The neurological manifestations of 21 patients at the time of diagnosis were dysarthria (78%), tremor (65%), drooling (58%), dystonia (55%), incoordination (45%), hypokinetic speech (42%), dysphagia (35%), myoclonus (30%), spasticity (15%) and psychiatric symptoms (10%).

Serum ceruloplasmin concentrations were low in 48 patients (73.8%) and urine copper values were raised in 45 patients (69.2%). The presence of K-F rings was observed in 35 of these patients (53.8%). The penicillamine challenge was performed in 42 patients (64.6%) and was >1,600 *μ*g/24 h in 25 patients (59.5%). Histological findings in the liver specimen included steatosis in 4 patients, chronic active hepatitis in 2 patients and cirrhosis in 2 patients.

MRI or CT findings of the brain were abnormal in all 26 symptomatic patients. The most frequently observed was increased density on CT and hyperintensity on T2 MRI in the region of the basal ganglia.

Seven of 15 patients carried the Arg778leu (CGG-CTG) mutation in the ATP7B gene, among whom 5 were homozygous and 2 were heterozygous.

### Clinical course

The mean duration of a combination of D-penicillamine and zinc, before stopping D-penicillamine, was 17.5 months (range, 6–28 months) and from that time the patients were treated with zinc alone. The median follow-up period for the group as a whole was 7.3 years (range, 6 months to 18 years).

### Liver function

The serum aspartate transaminase (AST) and alanine transaminase (ALT) levels were significantly decreased 6 months after treatment. Of the 55 patients with initial hepatic symptoms, 43 (78.2%) presented improved liver function during treatment, 4 (7.3%) maintained a stable hepatic condition and 6 (10.9%) presented decreasing liver function. Of these 6 patients, two were attributed to poor compliance with therapy and succumbed to hepatic failure. The other 4 patients presented decreasing liver function with progression to end-stage liver disease and successfully received a transplant.

### Neurological outcome

Improvement began a few months after initiation of therapy and was more evident in the first 2 years of treatment. No patient presented significant neurological deterioration.

### MRI

Follow-up MRIs were performed on 10 patients. Four patients demonstrated excellent recovery following treatment and presented almost complete resolution of hyperintense lesions on T2 in the basal ganglia. Residual abnormalities on MR images were present in 3 patients who made a complete clinical recovery.

### Copper and zinc metabolism

The 24-h urinary copper excretion levels were significantly lower after 2 months of treatment in comparison with the levels observed before treatment. We also monitored serum-free copper and the mean value significantly decreased from the baseline level (Table II). With zinc therapy, the plasma zinc and urine zinc levels increased significantly between baseline and after 1 year and was sustained in subsequent years of zinc therapy.

### Side-effects

Symptomatic patients were initially treated with a combination of D-penicillamine and zinc sulfate. However, in 6 (10.5%) patients, D-penicillamine was discontinued due to adverse effects (granulocytopenia in 2 patients, nephrotoxicity in 1 patient and eruption in 3 patients) that occurred within the first 12 months of treatment. No initial neurological deterioration was observed. Adverse reactions of large-dose zinc sulfate were minimal. Initial abdominal discomfort occurred only in 6 patients (9.2%) and disappeared after 1–2 months.

### Overall outcomes

The majority of patients (89.2%) had a stable or improved course of disease. One patient succumbed to the complications of variceal bleeding and 2 patients succumbed to hepatic failure. Four patients were treated successfully with liver transplantation.

## Discussion

Our study compiles data on 65 patients with WD and long-term observation is reflected in a mean follow-up of 16.5 years. In the present study, we demonstrated that a combination of low-dose D-penicillamine and high-dose zinc sulfate as initial therapy followed by a maintenance therapy of zinc sulfate alone controls WD effectively and safely. The majority of patients (89.2%) had a stable or improved course of disease. The patients received high doses of zinc without major side-effects. Side-effects from D-penicillamine were also less than has been reported in another series (12). Only 10.5% of the symptomatic patients were forced to stop D-penicillamine due to toxicity and they received zinc sulfate alone.

D-penicillamine is a controversial treatment for WD due to its reported side-effects and potential initial worsening of neurological manifestations. Wiggelinkhuizen *et al* (13) reported that 31.3% patients treated with D-penicillamine had severe side-effects, including neurological worsening, pancytopenia, nephrotoxicity, polyneuropathy, optic neuritis and polymyositis. Of the patients who worsen, 50% never recover. Therefore, it is considered that D-penicillamine should not be the drug of choice for patients with neurological symptoms. Brewer (14) recommended that it should not be used as initial therapy in WD. Currently, trientine is the recommended chelator for treatment of patients with hepatic WD (15). For the initial treatment of patients presenting with neurological disease, experts suggest the use of an experimental drug, tetrathiomolybdate (14). However, the experience with these drugs in China is very limited, due to the high cost and non-availability of the drug.

Numerous reports have been published concerning the use of zinc salt for treating WD (16,17). Zinc acetate was approved by the US Food and Drug Administration (FDA) in 1997 for the treatment of WD and quickly became the preferred treatment as a life-long maintenance therapy. Zinc acts by inducing intestinal and liver cell metallothionein, which binds to copper with a high affinity and prevents the transfer of copper into the circulation. Since copper also enters the gastrointestinal tract from saliva and gastric secretions, zinc treatment generates a negative balance for copper and thereby removes stored copper. The major advantages of zinc salt are its extremely low level of toxicity and low cost. However, zinc is too slow acting. It takes 4–6 months to control the toxic effects of copper. During this prolonged period of ongoing copper toxicity, the disease may progress on its own. The authors considered that copper removal therapy should be provided before zinc maintenance therapy for symptomatic patients. In our earlier study, we recommended a combination of low-dose D-penicillamine (8–10 mg/kg/day) and high-dose zinc sulfate as the initial therapy. The present study further proved the effectiveness and safety of this regimen. The usual or standard dose of D-penicillamine is recommended as 20 mg/kg per day in children. Due to frequent side-effects and initial neurologic deterioration of D-penicillamine therapy, we decreased the dose of this medicine. No D-penicillamine-induced neurological deterioration was noted on the initiation of treatment with D-penicillamine at a low dose and side-effects were observed in <11% of patients over the entire follow-up period. The recommended daily dose of zinc in children is 25 mg × 2 until age 6 years, 25 mg × 3 until age 16 years or until reaching a weight of 125 pounds and then the regular dose is 50 mg × 3 (18). Due to a large number of young children in the present study (7 patients aged <6 years and 25 patients aged <10 years), we selected an effective dose for the younger children. The daily dose of zinc sulfate used was 300–750 mg (68–170 mg elemental zinc) and the average amount was ≥550 mg (125 mg elemental zinc).

There are concerns with regard to a possible overtreatment with zinc. High doses of zinc have been reported to cause elevated serum lipase and amylase levels, as well as a reduction of high-density lipoprotein cholesterol in male patients, and even immune dysfunction in certain cases. However, these have been ruled out by well-designed studies (4,19). In our study, we did not observe any indication that our patients became more susceptible to infection and leukopenia. Copper balance, 24-hour urinary copper excretion and non-ceruloplasmin plasma copper concentration all indicated good copper control during zinc therapy. Six of the patients had low level urine copper and were temporarily withdrawn from the drug, starting back with a lower dose after 2–4 weeks. Although higher urinary zinc excretion and a higher concentration of plasma zinc was observed in the majority of children, no clinical side-effects other than slight gastrointestinal discomfort were observed.

In summary, the results of this study indicate the effectiveness of low-dose D-penicillamine and high-dose zinc sulfate as initial therapy of patients with WD. No D-penicillamine-induced neurological deterioration was noted on starting D-penicillamine at a low dose and side-effects were observed in <11% of patients over the entire follow-up period. Therefore, we negated the suggestion that D-penicillamine should not be the drug of choice for patients with neurological symptoms. The combined therapy is deserving of wider use due to its effectiveness, safety and affordability.

## Figures and Tables

**Table I:** Frequency of clinical and laboratory criteria used for diagnosis of patients with Wilson’s disease.

Type of presentation	Hepatic	Hepatic-neurologic	Neurologic	Total
Number of patients	44	15	6	65
Gender (male/female)	20/24	8/7	4/2	32/33
Age at presentation (years ± SD)	7.3±1.6	8.2±2.3	9.8±2.1	9.0±2.0
Range (years)	5–13	7–13	7–13	5–13
5–6 (n)	4	2	1	7
≤10 (n)	18	5	2	25
≤13 (n)	22	8	3	33
Kayser-Fleischer rings (+)	17	12	6	35
Low serum ceruloplasmin (<20 mg/dl)	30	14	4	48
High 24-h urine copper (>100 *μ*g)	29	11	5	45
Low plasma copper (<100 *μ*g/dl)	30	9	4	43

**Table II:** Main laboratory tests before treatment and during follow-up (mean ± SD).

Type of test	Initial	1 year	5 years	Normal values
24-h urine copper (*μ*g/24 h)	153.4±54.3	47.6±8.5^a^	37.1±16.7^a^	20–48
24-h urine zinc (*μ*mol/24 h)	3.8±2.0	16.5±5.9^a^	17.9±3.6^a^	2.3–18.4
Plasma zinc (*μ*mol/l)	8.1±3.9	25.8±6.3^a^	26.6±4.4^a^	8–23
Serum free copper (*μ*g/dl)	37.7±12.4	13.5±3.5^a^	8.5±3.4^a^	8–12
WBC (×10^9^/l)	7.3±1.8	6.9±1.4	6.4±2.1	4–10

a P<0.01, compared with baseline results. WBC, white blood cell.
